# Biocidal Resistance in Clinically Relevant Microbial Species: A Major Public Health Risk

**DOI:** 10.3390/pathogens10050598

**Published:** 2021-05-14

**Authors:** Elaine Meade, Mark Anthony Slattery, Mary Garvey

**Affiliations:** 1 Department of Life Science, Sligo Institute of Technology, Sligo, Ireland; garvey.mary@itsligo.ie; 2 Mark Anthony Slattery, Veterinary Practice, Manorhamilton, Leitrim, Ireland; markslatsvet@gmail.com

**Keywords:** resistance, risk, pathogens, environment, health, biocide

## Abstract

Antimicrobial resistance is one of the greatest dangers to public health of the 21st century, threatening the treatment and prevention of infectious diseases globally. Disinfection, the elimination of microbial species via the application of biocidal chemicals, is essential to control infectious diseases and safeguard animal and human health. In an era of antimicrobial resistance and emerging disease, the effective application of biocidal control measures is vital to protect public health. The COVID-19 pandemic is an example of the increasing demand for effective biocidal solutions to reduce and eliminate disease transmission. However, there is increasing recognition into the relationship between biocide use and the proliferation of Antimicrobial Resistance species, particularly multidrug-resistant pathogens. The One Health approach and WHO action plan to combat AMR require active surveillance and monitoring of AMR species; however, biocidal resistance is often overlooked. ESKAPE (*Enterococcus faecium*, *Staphylococcus aureus*, *Klebsiella pneumoniae*, *Acinetobacter baumannii*, *Pseudomonas aeruginosa*, and *Enterobacter* species) pathogens and numerous fungal species have demonstrated drug and biocidal resistance where increased patient mortality is a risk. Currently, there is a lack of information on the impact of biocide application on environmental habitats and ecosystems. Undoubtedly, the excessive application of disinfectants and AMR will merge to result in secondary disasters relating to soil infertility, loss of biodiversity and destruction of ecosystems.

## 1. Introduction

Antimicrobial resistance (AMR) is now recognised as a major public health crisis as essential antimicrobial drugs including antibiotics, antifungals, antivirals, antimalarials and anthelmintics [1] become less effective therapeutic options. Continued antimicrobial misuse and overuse in human and animal medicine, and poor prevention and control strategies have proliferated AMR and hurdled the planet into a post antibiotic era. The unwarranted prescription of antibiotics by general practitioners and veterinarians in the absence of diagnostic indicators, as metaphylactics, prophylactics and growth promotors, greatly proliferates AMR. Indeed, poor diagnostics, particularly when disease aetiology for bacterial, fungal, or viral infectious diseases is similar, encourages the misuse and overprescription of antibiotic agents [2]. The immense application of antibiotic agents in food production (agriculture and aquaculture) is also recognised as a major contributor to the emergence and proliferation of AMR. Globally, 100–200 thousand tonnes or 80% of antibiotics are used in food production annually [3], with an increase of 67% predicted by 2030 across all major livestock industries and aquaculture [4]. Europe has implemented bans on the use of growth-promoting antibiotics in food-producing animals, the United States and China, however, are more lenient, with 52% of antibiotics administered in China for growth-promoting activity alone [5]. Globally, AMR results in prolonged morbidity, increased mortality, economic burden, socioeconomic impacts and greatly hampers the success of Sustainable Development Goals, including the provision of maternal and child health, food security, poverty reduction and economic growth [6]. Methicillin-resistant *Staphylococcus aureus (MRSA)*, for example, is the most common Gram-positive multidrug-resistant (MDR) pathogen causing morbidity and mortality globally [7]. *Candida auris* is an emerging multidrug-resistant nosocomial fungus and is a major threat in healthcare settings [8]. Moreover, global disease outbreaks are becoming a constant threat, as is evident by the emergence of the highly pathogenic human coronaviruses, including SARS-CoV-2 (COVID-19), SARS-CoV-1 (SARS) and the Middle East respiratory syndrome (MERS-CoV). Studies report that COVID-19 can survive and remain infective for approximately 9 days on surfaces [9], making it highly transmissible.

As global initiatives push for research and development into novel antimicrobial agents for use as stand-alone or combination therapy options, there is also a need to establish strategies and preventative measures to reduce AMR. Effective disinfection and sanitation strategies are key in preventing communicable disease transmission in both human and animal environments. Biocides, which are chemicals used as sanitizers and disinfectants, consist of specific formulations containing one or more active ingredients that nonspecifically and fatally target microbial species. Typical commercial biocides used in clinical, industrial and domestic settings consist of quaternary ammonium compounds (QACs), benzalkonium chloride (BAC), chlorine and chlorine-based derivatives, acid anionic agents, hydrogen peroxide (H_2_O_2_), biguanides (chlorhexidine and alexidine), amphoteric surfactants, bisphenols (triclosan), alcohol, isopropyl alcohol (IPA), aldehydes (e.g., glutaraldehyde), iodine-releasing agents (iodophors), isothiazolones and peracetic acid [10]. As antimicrobial therapeutics become progressively less reliable, there is increasing pressure on effective disinfection protocols to prevent disease transmission in all areas where infectious diseases are a risk. A failure in these protocols will significantly impact on morbidity and mortality globally. The impact of biocidal use on AMR in species is under question however, as evidence suggests biocidal resistance, AMR and MDR mechanisms are interlinked. This review examines the association between biocidal use, biocidal resistance and antimicrobial resistance in clinically relevant species. The authors aim to explore how the application of biocidal agents in various settings promotes the joint emergence of biocidal resistance and AMR. 

## 2. Antimicrobial Biocide Use

In the European Union, disinfectants are classified as biocidal products regulated by the Biocidal Products Regulation (BPR) (EU) No 528/2012, ensuring efficacy and safety prior to marketing. Disinfectants can be classified into four overlapping categories including sanitizer, general disinfectant, sporicide and sterilant. Disinfectants, sanitizing agents and cleaning chemical agents have been used to inhibit and prevent microbial growth in pharmaceutical and medical device industries, healthcare, food, drinking water and domestic settings for decades. Effective cleaning and disinfection strategies are enforced to prevent disease transmission and control infectious disease by sanitising surfaces, fomites and personnel. In terms of disinfection, there are differences between disinfectants, sanitizers, antiseptics and sterilizing agents based on the desired objectives, the composition and concentration of the biocide, the contact time, residual levels and the area being disinfected [11]. In healthcare settings, the requirement for disinfection is determined by the nature of the item in terms of patient care. Medical devices are categorised as critical, semicritical and noncritical in terms of the risk of transmission of infectious diseases to patients. Critical items, including implants, must be purchased sterile or steam-sterilised, whereas high-level chemical disinfectants glutaraldehyde, hydrogen peroxide, ortho-phthalaldehyde (OPA), peracetic acid with hydrogen peroxide, and chlorine are suitable for semicritical items such as endoscopies [12]. Noncritical items that only come in contact with skin require disinfection with low-level disinfectants such as QACs. In food production, disinfectants used in animal settings are strong, and often toxic biocidal chemicals are applied to contaminated surfaces, whereas biocides used in food processing and domestic environments are usually less toxic and more diluted. To achieve microbial death using biocidal solutions, cleaning must precede treatment to eliminate organic and inorganic material. Additionally, specific guidelines for chemical concentration, contact time, temperature and pH must be adhered to. Disinfectant efficacy is impaired by interfering substances, typically organic matter, temperature, pH, contact time and the concentration. For instance, the pH affects the reaction kinetics of the disinfectant and thus influences the antimicrobial activity by altering the disinfectant molecule or cell surface. Hence, while an increase in pH will improve the antimicrobial activity of certain disinfectants, including, QACs and glutaraldehyde, it will decrease the activity of others, such as iodine, hypochlorite and phenols. In addition, many disinfectants work optimally at higher temperatures (typically 20 °C), where a lower temperature can lead to loss of disinfectant efficacy, particularly for QAC and aldehyde-containing disinfectants [13]. On the other hand, oxidising agents such as chlorine- or iodine-based disinfectants are not as affected by low temperatures [14]; however, they are more prone to inactivation by organic matter. Importantly, alcohol-based disinfectants are not significantly hindered by the presence of organic matter contamination [15], unlike many other disinfectant types. Unlike antimicrobial therapeutics that specifically target microbial cell components, such as cell walls, specific enzymes and genetic material, biocides interact nonspecifically with microbes, having multiple targets [10] and varying efficacies dependant on the target microorganism. For example, QACs disrupt the lipid bilayer structure of cell membranes, leading to membrane destabilisation, loss of function/structure and cytoplasmic leakage. Consequently, vegetative bacterial and fungal cells, and enveloped viruses are most affected, where QACs are ineffective against nonenveloped viruses and spores. Moreover, Gram-negative bacteria are less affected by these agents, due to the presence of their outer membrane and glycolipid endotoxin component, when compared to that of Gram-positive species. In addition, higher concentrations of QACs are generally required to be effective against yeasts and mould species. On the other hand, oxidising agents such as iodine and chlorine exert a broader spectrum of activity, being active against bacteria (including recalcitrant Gram-negative pathogens), fungi and viruses. Indeed, biocides often differ in their relative efficacies against the myriad of microorganisms, mainly due the biocidal formulation, the efficacy of the active component, the use and contact time, and the adsorption and uptake by cells (where chemical composition and architectural structure vary among different microbes). Intracellularly, biocides cause cell damage by disrupting metabolic processes, coagulating cellular components, and disrupting proteins and/or genetic material [16]. The antimicrobial activity of biocides is either through growth inhibition (bacteriostatic and fungistatic) or as a killing agent (sporicidal, bactericidal, fungicidal and virucidal). As mentioned, susceptibility to biocidal activity varies amongst microorganisms and typically follows the order from least to most susceptible: prions, coccidia, endospores, mycobacteria, Gram-negative bacteria, fungal species and Gram-positive bacteria [17]. Biocidal activity against viruses depends on their structure, specifically on the presence of an envelope, where enveloped viruses are more sensitive than nonenveloped viruses [18]. To ensure efficacy, testing of disinfectants to determine antimicrobial activity via suspension tests such as the European standards EN 1276, 1650 and 1656 (amongst others) are conducted. These tests generally require a 5-log reduction of viable cell numbers within a set number of minutes [19]. Nonetheless, suspension tests do not mimic the growth conditions of microbial species present in environmental samples, do not assess microbial growth phases such as log or stationary phases and do not account for resistant species. The EN 13,697 is a surface test to determine efficacy on varying surface materials but does not account for biofilm formation. The use of biocidal solutions at subtoxic concentrations, times or other treatment parameters leads to the survival of subpopulations of microbial species. This selective pressure promotes biocidal resistance, which is becoming increasingly recognised as a risk to public health safety, particularly when observed in species displaying multidrug resistance to antimicrobial therapeutics. Of greatest concern is the promotion of therapeutic resistance following exposure to biocidal solutions, termed cross-resistance [20].

## 3. Biocidal Resistance

The emergence of disinfectant-resistant microbes raises many issues, from disease transmission in healthcare settings and food production, to the manufacture of sterile pharmaceutical drugs and medical devices. The definition of biocidal resistance remains somewhat uncertain, some suggest resistance is a decrease in susceptibility as determined by an increase in the minimum inhibitory concentration (MIC) while others suggest bacteria surviving biocidal exposure at any usable concentration are deemed resistant [20]. 

### 3.1. Bacterial Biocidal Resistance

In 2017, the World Health Organisation (WHO) announced a list of pathogens urgently requiring new antimicrobial options, including the ESKAPE pathogens, namely, *Enterococcus faecium*, *Staphylococcus aureus*, *Klebsiella pneumoniae*, *Acinetobacter baumannii*, *Pseudomonas aeruginosa* and *Enterobacter* species, which are now designated priority pathogens [21]. These nosocomial pathogens are responsible for approximately 400,000 morbidities and 25,000 mortalities in Europe and approximately 2 million morbidities and 23,000 mortalities in the United States, annually [2]. Studies describe biocidal resistance in many of these clinical species, particularly *Pseudomonas*, *Acinetobacter*, and *Staphylococcus* [22]. Similar to antibiotic resistance, biocidal resistance is also intrinsic, acquired via gene mutations or transmitted on plasmids via horizontal gene transfer (HGT). Intrinsic resistance is related to membrane structure, efflux pumps or formation of endospores and biofilms. Gram-negative species such as *E. coli*, *Klebsiella*, *Proteus* and *Pseudomonas* are also less permeable to biocides due to the presence of an outer membrane and lipopolysaccharide layer [16]. Bacteria can modify their membrane, upregulate efflux pumps and initiate biofilm formation in response to subtoxic biocide exposure and residual disinfectant concentrations. Resistance is acquired via the sharing of BRGs via HGT on plasmids and is believed to be the link between biocidal resistance and AMR in species. Biocidal resistance genes (BRGs) have been identified in many bacterial species, including the *qacE* and *qacA/B* genes common in the Enterobacteriaceae family and *Pseudomonas* and *qacA/B* genes in *S. aureus* conferring resistance to QACs [22]. The *qac* genes code for nonspecific efflux pumps that are active in removing biocidal agents from bacterial cells. There are five classes of efflux pump: (1) ATP (adenosine triphosphate)-binding cassette (ABC) family, (2) the major facilitator superfamily (MFS), (3) the resistance/nodulation/division (RND) family, (4) the small multidrug resistance (SMR) family and (5) the multidrug and toxic compound extrusion (MATE) family [17]. The expression of efflux pumps following exposure to biocides can be induced by affecting global gene regulators, particularly marA and soxS [19]. Studies assessing the expression of efflux pumps following exposure to triclosan show that high-level resistance was associated with efflux activity [23]. A high prevalence of efflux pump genes (*qacA/B*, *norA/b* and *smr*) was found in species demonstrating biocidal resistance isolated from environmental hotspots laden with biguanides and QACs [24]. ABC pump EfrAB is seen in *Enterococcus*, *Staphylococcus* and *Bacillus* species conferring resistance to chlorhexidine and triclosan. MATE pumps have been identified in many species, including *Pseudomonas*, *Vibrio*, *Acinetobacter*, *Proteus*, *Neisseria* and *Staphylococcus*, conferring resistance to benzalkonium chloride, triclosan and chlorhexidine [17]. The RND family of efflux pumps are more commonly found in Gram-negative species having broad-spectrum activity expelling antibiotics and biocides, including fluoroquinolones, β-lactams, tetracycline and linezolid [25], whereas MFS pumps such as NorA are commonly found in Gram-positive species, including *S. aureus*, PmrA in *S. pneumonia* and EmeA in Enterococcus, conferring MDR and biocide resistance [26]. In Gram-positive species such as *S. aureus*, efflux pumps are plasmid-encoded, such as the SMR pumps and the MFS QacA/B efflux pumps. In Gram-negative species, efflux pumps are often chromosomally encoded and are also multidrug pumps [25]. The RND efflux pump, MexCD-OprJ, found in Gram-negative species confers resistance to fluoroquinolones and is inducible by exposure to QACs [25]. It must be noted that efflux pumps also provide resistance to bile in enteric species, allowing pathogen colonisation, virulence, biofilm formation and survival in the host [27]. Studies have also demonstrated that exposure to chlorhexidine upregulated vancomycin and daptomycin resistance genes in *E. faecium* [28] and subtoxic exposure of *P. aeruginosa* and *S. aureus* to QACS and amphoterics promoted AMR in these species [19]. AMR outbreaks caused by *Burkholderia cepacia* associated with antiseptic chlorhexidine wipes in neonatal and paediatric wards have been reported, with *Achromobacter spp*. infections associated with contaminated didecyl diammonium chloride solution [14]. Biocidal resistance has been identified in extended-spectrum beta-lactamase (ESBL)-producing Enterobacteriaceae where 100% were found resistant to chlorhexidine and 80% to BACs, where the *qacEΔ1* gene (Table 1) was detected [29]. ESBL Enterobacteriaceae infections are increasing globally and are recognised as a major health crisis where community- and hospital-acquired infections result in potentially fatal bacteriemia amongst other disease states [30]. Unlike antibiotic resistance, resistance to biocides via target alteration is not common, as biocides typically kill via a multi-hit process. However, mutations in the FabL gene, which is responsible for fatty acid synthesis, have been detected in *E. coli*, *P. aeruginosa*, *Staphylococcus* species and *A. baumannii* where resistance to triclosan was evident [17]; triclosan is a reversible inhibitor of FabL. Microbial biofilms are organized communities of cells that secrete an extracellular polymer matrix (EPS) enabling adherence to biotic (living) and abiotic (non-living) surfaces [31]. Biofilms are the natural state of bacterial cells (sessile), as opposed to planktonic cells, and are believed to be associated with 80% of human infections such as pneumonia in cystic fibrosis patients, chronic otitis media and implant- and catheter-associated infections [32]. Biofilm formation on abiotic and biotic surfaces greatly reduces the permeability of antibiotics and biocide solutions, ensuring the survival of the biofilm community. Additionally, studies demonstrate that multispecies biofilms are more biocidal resistant than single species, where *P. aeruginosa* and *K. pneumonia* mixed biofilms demonstrated resistance to clinical concentrations of chlorhexidine and H_2_O_2_ [33]. Biocidal efficacy against biofilms varies amongst disinfectants, with peracetic acid more effective against *A. baumannii*, *K. pneumoniae* and *P. aeruginosa* biofilms [34].

### 3.2. Fungal Biocidal Resistance

Fungal species exists as multicellular, threadlike, cylindrical structures termed hyphae, which also form mycelia, producing macroscopic mushrooms [50]. Some fungi termed dimorphic fungi may also exist as single cells known as yeasts. Antifungal resistance is a major concern as more than 300 million people suffer fungal infections yearly across the globe, resulting in approximately 1,350,000 deaths [51], particularly in immunocompromised patients. Approximately half a million people suffer from candidiasis alone globally, with a mortality rate of 45–75% annually [52]. Clinical fungal species such as *Candida*, *Cryptococcus* and *Aspergillus* are a major concern as they demonstrate resistance to numerous drug therapies such as fluconazole, amphotericin and caspofungin [53], where biocidal resistance may also be evident. MDR in clinical isolates as observed in *C. albicans*, *C. glabrata* and *C. auris* can be intrinsic or acquired. *Candida krusei* and *C. auris* are intrinsically resistant to fluconazole, whereas *Cryptococcus* species are intrinsically resistant to caspofungin [54]. Acquired resistance is a result of prolonged exposure to antifungal therapeutics where subtoxic concentrations of biocides may also induce resistance. In fungal species, the development of AMR is resultant from similar mechanisms as those in bacterial species, including altering target proteins/enzymes, efflux pumps, altering membrane permeability/drug uptake and biofilm formation [55], and is regulated by resistance genes. Fungal species also make spores as part of their reproductive life cycle; however, these are less biocidal resistant than bacterial spores [56]. Fungal efflux pumps are major contributors to drug and biocidal resistance in yeast (*Saccharomyces* species) and fungal species (*Aspergillus, Neurospora* and *Cryptococcus* species). Efflux pumps are abundant in fungi and yeast as they are vital for nutrient uptake, homeostasis, secretion of secondary metabolites (including antibiotics) and the efflux of toxins and chemicals [50]. The ABC and MFS efflux families are found in fungal species conferring resistance to antifungal therapeutics [57] and biocides. In clinically relevant fungal species, including *Candida*, increased expression of membrane transporters and efflux pumps (*CaCDR1* and *CaCDR2*) correlates with resistance to azole antifungals [58]. Differences in susceptibility amongst fungal species may also relate to variations in their cell wall, for example, dematiaceous fungi contain melanin in their cell wall, which may confer resistance to biocidal agents [59]. Studies have described the efficacy of some biocides against psychotropic fungal and yeast species, where resistance to QAC and formaldehyde was evident. Furthermore, osmophilic yeast was also inactivated following exposure to formaldehyde and peracetic acid [60]. While studies examining the efficacy of peracetic acid against a range of *Candida*, *Trichosporon* and *Rhodotorula* species determined that an exposure time of up to 60 min was required for cell death [60]. The BSEN 13,624 and 1275 standards are the efficacy tests for fungicidal and yeasticidal activity in medical areas, evaluated using *Candida albicans* ATCC 10,231 requiring a 4-log reduction in 60 min for disinfectants. Studies have demonstrated that BACs are ineffective against planktonic *Candida* species according to EN 1275 [61], with QACs only weakly active against planktonic cells of *Candida* species [62]. Cadnum et al. also demonstrated that H_2_O_2_-based disinfectants are effective against *Candida* species, including the nosocomial *Candida auris*. A 1% sodium hypochlorite solution demonstrated efficacy against *Candida* species, in both planktonic and biofilm forms, with 0.1% giving a 4.5-log inactivation of *C. auris* in 5 min [63]. The concentrations of H_2_O_2_, ethanol and sodium dodecyl sulphate required to kill *Candida* biofilms biocides must be several folds higher than the concentration effective for planktonic cells [64]. A 2% chlorhexidine gluconate hand sanitiser failed to eradicate *C. auris* within 2 min, whereas it passed the EN 13,624 test for *C. albicans* ATCC 10,231, thereby demonstrating the failure of EN testing methods to show efficacy against clinical strains [63]. Studies by Sisti et al., 2012, report that chlorine and peracetic acid concentrations up to 10 ppm failed to inactivate *Aspergillus* in water and concluded that *Aspergillus* species are highly resistant to both biocides even when in a combined solution [65].

### 3.3. Viral Biocidal Resistance

Viral susceptibility and resistance to disinfectants is predominately related to the presence of an envelope, where three types exist: enveloped viruses, large nonenveloped viruses, and small nonenveloped viruses. Small, nonenveloped viruses such as noroviruses and picornaviruses are more biocidal resistant, followed by large nonenveloped viruses such as papillomaviridae. The lipid envelope present on enveloped viruses (hepatitis B, HIV, herpes virus and SARS-CoV) is required for host cell infectivity, whereas nonenveloped viruses (polio and hepatitis A) use a protein coat for this purpose [66]. As with other microbial species (bacteria and fungi), viral inactivation is related to disruption of the cell structure, protein coagulation and/or protein denaturation [13]. However, virus inactivation is complex as highly related virial families display varying susceptibility to the same biocide, for example, poliovirus type 1 (Bruhilde) is twice as resistant to chlorine as poliovirus 1 Mahoney [67]. Studies also demonstrate that viral aggregation and particle association enables biocidal resistance, whereas dispersed viruses appear more sensitive [68]. A loss or reduction in viral infectivity as determined by carrier and suspension tests is the measure of disinfection efficacy. As with all microbial species, key biocidal parameters impact on biocidal efficacy, including contact time, concentration, environmental conditions (pH and temperature) and the target species. For enveloped viruses, lipophilic disinfectants such as the QACs may be effective, whereas nonenveloped species require the destruction of the viral capsid proteins and glutaraldehyde or sodium hypochlorite appears suitable for use [13]. Studies have shown that ethyl alcohol proved effective at inactivating enveloped viruses including herpes and influenza and some nonenveloped viruses (adenovirus and rotavirus), wherase IPA was effective against enveloped but ineffective towards nonenveloped viruses [69]. IPA is lipophilic in comparison to ethanol, which may explain its efficacy towards enveloped viruses. Studies also demonstrate that SARS-CoV1 is sensitive to commercial disinfectants including peracetic acid, ethanol 70%, sodium hypochlorite and chlorhexidine digluconate, whereas influenza displays resistance to chlorhexidine digluconate and BAC [70]. The failure of BAC to inactivate this nonenveloped virus is not surprising as it is a quaternary ammonium compound. Amphiphilic surfactants containing both hydrophilic and lipophilic segments are effective at inactivating viruses due to their dual water and fat solubility. Lipophilic regions are effective against enveloped viruses including SARs-CoV1 and SARS-CoV2, and the hydrophilic region is effective against nonenveloped viruses via alteration of protein moieties [71]. Small nonenveloped viruses, including noroviruses, are typically more resistant to disinfectants (Table 2); therefore, oxidizing agents including hydrogen peroxide, and peracetic acid and sodium hypochlorite are recommended [13]. Ethanol and IPA between 70% and 90% at an exposure time of 30 s is effective against SARS-CoV, whereas H_2_O_2_ requires 1 min at 1–3% concentration and aldehydes require 2 min exposure to 3% [72]. Povidone-iodine, which is commonly used as a skin, nasal and oral cavity disinfectant, has demonstrated good efficacy against SARS-CoV-2 and MERS-CoV even in soiled conditions [13]. To achieve complete inactivation of SARS-CoV-2 with chlorine dioxide however, a concentration of 20 ppm for 5 min was required in wastewater, where a 10 ppm solution only achieved a 55.3–68.4% inactivation [73].

## 4. Clinical Impact of Antimicrobial Resistance

The purpose of disinfection in clinical, veterinary, domestic and medical sectors (medical and pharmaceutical) is to reduce the viable microbial load on surfaces and fomites that are directly responsible for pathogen transmission. Biocidal efficacy, however, is impacted by the presence of interfering substances, typically, organic matter, temperature fluctuations, pH, contact time and the concentration applied. The spread of infectious diseases where AMR pathogens often result in patient mortality represents a serious public health risk. The presence of biocidal resistance in AMR species represents an increased risk where disease transmission may not be preventable. The presence and mechanisms of biocidal resistance have not been elucidated for many disinfectants and clinically relevant species. There is also a lack of detailed information on which biocidal agents are more prone to inducing AMR in species than others. Currently, there are numerous zoonotic pathogens transmissible to humans via direct animal contact or food contamination, including AMR species of *Cryptococcus*, *Candida*, *Aspergillus*, *Campylobacter*, *Listeria*, *Salmonella*, *E. coli O157*, *Vibrio*, *Clostridium* and *Streptococcus* [79], which, like the nosocomial ESKAPE pathogens, display antibiotic and biocidal resistance [80]. For example, studies have described antibiotic-resistant clinical *E. coli* strains that require higher concentrations of BAC for disinfection, and foodborne *Pseudomonas* strains demonstrating resistance to BAC and ampicillin, amoxicillin, erythromycin and trimethoprim [81]. These Gram-negative aerobic bacilli are the main pathogens associated with nosocomial (hospital-acquired) infections, including pneumonia, bacteraemia and UTIs, and are particularly associated with infectious disease in intensive care units [82]. Morbidity rates of 61% for *Pseudomonas* [83] and 11.5% for *E. coli* [84] apply. Moreover, sublethal exposure of the zoonotic *Salmonella typhimurium* to QACs promoted resistance to chloramphenicol, tetracycline, ampicillin and acriflavine [85]. *Salmonella* species showing resistance to sodium hypochlorite have displayed resistance to ceftazidime (*S. enteritidis*) and amikacin, tobramycin, cefazolin and cefotaxime in *S. typhimurium* [81]. The CDC estimates that *Salmonella* results in 1 million cases of infectious diseases yearly in the US and is the second most common foodborne pathogen in Europe (after Campylobacter). The incidence of nosocomial fungal infections associated with treatment failure is increasing, globally. Invasive fungal pathogens, including *Cryptococcus*, *Candida* and *Aspergillus*, result in 90% of life-threatening fungal disease in immunocompromised persons [51]. *Candida auris,* an emerging nosocomial MDR fungus, was responsible for 50 and 33 cases of disease in the UK and Spain, respectively, in 2016 [86], where *C. auris* has a 30-day mortality rate of 35%. There is a lack of information specifically detailing the susceptibility of clinically relevant fungi to common disinfectants or detailing mechanisms of resistance present. Zoonotic fungal infections, including dermatophytosis, sporotrichosis and histoplasmosis, are an important public health issue globally, however there is a lack of information on adequate preventative measures to control transmission [87]. Similar to bacterial species, the presence of fungal biofilms allows microbial species to persist in the environment and resist disinfection solutions. Currently, there is a lack of information on the susceptibility of fungal biofilms and multispecies biofilms to disinfection regimes. Many viruses, including hepatitis B and C, rotavirus, enteroviruses and cytomegalovirus, are associated with nosocomial transmission. Respiratory viruses, including respiratory syncytial virus, adenovirus, rhinoviruses, SARS-CoV-2 and influenza, are the main nosocomial viruses where direct contact between patients, healthcare staff, fomites and air and water droplets promotes transmission where they can cause or contribute to patient mortality [88]. Studies indicate that children are more susceptible to nosocomial viruses, with 49% of viral infections occurring in premature infants, while 24% of nonventilated pneumonia was viral in nature [89]. Of influenza cases in hospitals, 5.65% are related to nosocomial transmission and result in chronic illness and mortality. Preventative measures, including suitable disinfection regimes and parameters ensuring viral inactivation or evidence of resistance, are also essential. 

To prevent nosocomial transmission, effective infection control systems that are heavily reliant on disinfection control measures must be in place. To be effective in a clinical setting, disinfectants must demonstrate efficacy against a broad range of microbial pathogens from bacterial, fungal and viral species. A “one fits all” disinfection solution is not realistic however, as variations in environmental factors and microbial species will impact efficacy. Antiseptics used clinically for skin disinfection often contain alcohol or IPA, with newer solutions containing additional agents such as chlorhexidine, povidone iodine or benzalkonium chloride. The added benefit of these additional biocides is uncertain however, and no added efficacy has been demonstrated for BAC or povidone [81], and BAC runs the risk of inducing AMR in species. While the emergence of antimicrobial resistance in microbes may become evident due to a lack of response to drug therapy, the emergence of biocide resistance can go unrecognised indefinitely. In 2015, the WHO announced its Global Action Plan aiming to combat AMR, which included limiting the application of numerous critically important antibiotics in veterinary applications. Perhaps a focus on the correct use and optimal application of key biocidal solutions must also be considered, particularly in clinical and veterinary settings where disease transmission is high. The safety implications of the misuse and overuse of disinfectants must also be considered, as certain disinfectants (sodium hypochlorite, sodium chloride, chlorine and QACs) are irritants and corrosive to the respiratory and intestinal mucous membranes of humans and animals [90], where chlorine is carcinogenic. Currently, there are no comparable guidelines in place for monitoring the use of disinfectants on a large scale [91] in terms of environmental safety.

## 5. Environmental Impact

Antimicrobial agents, including disinfectant solutions, AMR species and biocide resistance genes, are present in the environment in hotspot locations associated with agriculture, aquaculture and hospital wastewater. Urban water effluents from wastewater treatment plants are among the main anthropogenic sources of antimicrobials and biocides in the environment. Alarmingly, estimates show that approximately two-thirds of global rivers, surface waters, groundwater and wetlands are contaminated with micropollutants, including antibiotics and biocides. This contamination has a negative impact on ecosystems and biodiversity [92]. Environmental risks of antimicrobial pollution include residue accumulation, a loss of biodiversity, the selection and proliferation of resistance genes in species and the emergence of MDR [93]. The presence of biocides and biocide resistance genes also impacts the microbiota of soil and water and can be detrimental to soil fertility and other ecological functions. Soil fertility and health are critically important to biodiversity, all biotic life and food production. Protecting soil biodiversity and fertility is vital to achieving Sustainable Development Goals relating to food, health, water and climate where the impact of biocidal contamination on soil microbiota must be established. Anthropogenic application of antimicrobials has a huge impact on the environmental resistome, altering the natural microbiome present. More importantly, the presence of different resistance genes (biocide and antibiotics) in the environment encourages their combination into the same genetic element (plasmids and integrons), which can then be transmitted between species via HGT [94]. Currently, data is lacking on the minimal biocide concentrations inducing resistance and promoting HGT between species. The toxicological effect of biocide pollution on ecosystems also warrants investigation as many disinfectants and their by-products are environmental toxicants. At present, this information on the impact of biocides and biocide-resistant microbes in the environment is severely lacking. For example, chlorine-based disinfectants are toxic to birds and mammals and can bioaccumulate in the food chain. Studies report on the death of hundreds of birds from 17 species in China resultant from disinfectant overuse relating to COVID-19 [91]. Studies have also reported that the chlorination of water is associated with promoting the emergence of highly tetracycline-resistant *E. coli* strains [95]. *E. coli* is abundant in the environment as an enteric species of numerous animals and is highly prone to HGT, sharing mobile genetic elements. Studies have detected QACs in surface and wastewater effluent at concentrations up to 60 μg/L and at greater concentrations in influent wastewater [96], where they are toxic to numerous environmental species, including fish, algae and daphnids [97]. The excessive use of disinfectant products with subsequent accumulation in the environment can lead to secondary disasters to terrestrial and aquatic ecosystems. The WHO Action Plan objective 4 (optimise the use of antimicrobial agents) considers the role of the environment in AMR and details the need to develop standards and guidance for antimicrobial agents in the environment. A holistic AMR action plan must incorporate environmental factors including biocidal pollution, transmission of biocidal resistance genes and species to effectively impact on antimicrobial resistance, globally.

## 6. Conclusions

To safeguard human and animal health, reduce antimicrobial use and promote environmental safety, the widespread use of antimicrobials needs to be reduced in all sectors. Many studies now report on the relationship between biocide resistance and AMR, highlighting the need for better biocidal application. Resistant microbes and resistant genes can and do disseminate within the environment and between humans and animals, and so the holistic One Health approach must be applied. As part of One Health and the WHO Action Plan, reducing antibiotic use in animal production is key; however, it requires investment on a large scale. While the EU banned the use of antibiotics as growth promoters in food-producing animals in 2006, there is still illegal and unregulated use happening globally. Research and knowledge transfer into sustainable production practices such as vaccination programmes, hygienic animal husbandry, effective biosecurity practices and optimal animal nutrition to decrease disease prevalence are needed. With the emergence and re-emergence of infectious diseases, there comes a global increase in the use of antimicrobials including disinfectants. These biocides ultimately enter waterways and can be transported through soil, surface water, or groundwater, where they impact natural ecosystems. It is essential to determine the impact of this on the environment, and animal and human health long term. Otherwise, there is a global risk of secondary disasters relating to irreversible damage to ecosystems, biodiversity and natural habitats. It is important to consider biocide resistance genes such as antimicrobial resistance genes as emerging contaminants due to their environmental mobility and dissemination. Currently the resistance mechanisms for many commercial biocides are unknown; however, this information is essential to determine if resistance emergence and selection is going to occur. Information on biocide-specific resistance and its relationship to broad antimicrobial resistance is needed. Filling these knowledge gaps is vital to ensure effective disinfection protocols, particularly in times of endemic and pandemic disease outbreaks. 

## Figures and Tables

**Table 1:** Classification of beta-lactamase enzymes associated with ESBL activity in clinically important pathogens where biocidal resistance has been detected. Enzyme inhibitors to overcome AMR are also listed.

	Enzyme Type	Representative Enzymes	Known Substrates	Inhibitor Profile	Clinically Associated Pathogens	Biocidal Resistance
**Serine β-lactamases**	Penicillinase	PC1/*blaZ*	Penicillins	CA and TZ	MRSA	*qacA/B* (acquired), *norA* and *lmrS* (intrinsic) genes encoding MFS pumps. *MecA* (MATE superfamily) and *sepA* multidrug efflux pump genes. SMR pumps encoded by *smr* (also known as *qacC/D* and *Ebr*), *qacG*, *qacH* and *qacEΔ1* (acquired) [35,36,37]
	Broad- spectrum (TEM, SHV-type)	TEM-1, -2 and -13, SHV-1 and -11	Penicillins and 1st-generation cephalosporins [38]	CA, TZ and SB	*Enterobacteriaceae (E. coli, K. pneumonia, Proteus sp.) non fermenters (i.e., Pseudomonas aeruginosa., Acinetobacter baumannii) and Neisseria gonorrhoeae*	Acquired efflux resistance to QACs and chlorhexidine encoded by *qacEΔ1, qacE, qacG, qacH* and *emrE* (SMR), *qacA* (MFS) and *cep A* genes common in many Enterobacteriaceae [39,40] and non-fermenters [41,42] Multidrug efflux MATE pumps (chromosomally encoded) conferring resistance to biocides and antimicrobials, examples include YdhE of *E. coli*, PmpM of *P. aeruginosa*, and AbeM of *A. baumannii* [43] Upregulation of chromosomally encoded RND pumps conferring cross-resistance to biocides, antimicrobials and other agents (dyes, metals), examples include AcrAB-TolC, AcrEF-TolC in *E. coli* and other Enterobacteriaceae [39] MtrD in *N. gonorrhoeae* [44] MexAB-OprM, MexCD-OprJ, MexEF-OprN and MexJK pumps in *Pseudomonas* [45] AdeABC, AdeFGH, AdeIJK and AbeD efflux systems in *A. baumannii* [46]
		TEM-30 and -31, SHV-10	Penicillins	Reduced binding to CA or inhibitor resistant apart from AV		
	ESBL (TEM, SHV, PER, VEB, CTX-M-type)	TEM-3, and -10, SHV-3, CTX-M-1, -14, -15 and -44, PER-1, VEB-1	Penicillins, 1st, 2nd- and 3rd-generation cephalosporins and monobactam	CA, TZ, SB and AV		
		TEM-50 and -158		Reduced binding to CA or inhibitor resistant apart from AV		
	Carbenicillinase	PESE-1, -3 and -4, CARB-1	Penicillins and carbenicillin	CA, TZ and SB		
	Carbapenemase (KPC, GES, SME-type)	KPC-2 and -10, IMI-1, SME-1, and -2, GES-2 and -7	All beta lactams	Variable to CA, TZ and AV	*P. aeruginosa,* and *K. pneumonia* (and other *Enterobacteriaceae)*	
	OXA-type (Broad spectrum, ESBL and Carbapenemase)	OXA-1, OXA-9, OXA-10, OXA-2 [38]	Penicillins (oxacillin, cloxacillin)	Variable to CA, TZ and AV	*Enterobacteriaceae (K. pneumonia, E. coli, Enterobacter sp.), nonfermenters and Neisseria gonorrhoeae*	
		OXA-11, OXA-14, OXA-15,	Penicillins, 3rd-generation cephalosporins, monobactams			
		OXA-3, OXA-51, OXA-58, OXA-23, OXA-48	All beta lactams/carbapenems			
	AmpC cephamycinases	AmpC (chromosomal encoded)	All beta lactams except carbapenems	Inhibitor resistant apart from AV	*Citrobacter, Serratia, Enterobacter* spp., and *P. aeruginosa* (expression usually inducible) and Enterobacteriaceae (not as inducible)	Studies report on the presence of efflux pumps belonging to the MATE and RND families in *Enterobacter*, where AmpC is inducible in these species [47] *qacE∆1* is commonly reported in enteric pathogens, being associated with class 1 integrons that carry multiple gene cassettes including AmpC β-lactamases [48]
		MOX, ACC, FOX, DHA, CMY, MIR-type (plasmid encoded)			Non fermenters and Enterobacteriaceae	
**Metallo-β-lactamases**	Carbapenemases (IMP, VIM, NDM-type)	IMP-1, VIM -1 and -2, NDM-1 [38]	All beta lactams except aztreonam	EDTA or 1-10 phenanthroline, mercaptopropionic acid or sodium mercaptoacetic acid and dipicolinic acid	*Pseudomonas and Acinetobacter* sp.	RND efflux pumps on plasmids that carry resistance determinants such as *blaNDM-1* have been reported [41,49] Association of *qac* genes with the presence of NDM, VIM and IMP beta lactamases reported in clinical *A. baumanii* [42]

CA—clavulanate acid, TZ—Tazobactam, SB—Sulbactam, AV—Avibactam.

**Table 2:** Clinically important fungal and viral pathogens and associated antimicrobial and biocidal resistance.

	Medically Important Pathogen	Associated Disease	Antimicrobial Resistance	Biocidal Resistance
Fungal	*Candida albicans*	Candidemia, mucosal candidiasis, cutaneous infections	Mutations in ERG11 and Upc2p, and overexpression of Cdr1, Cdr2 and Mdr1 confer azole resistance Polyene resistance is linked to changes in ERG3 and ERG6 Mutations in *CaFKS1* confer resistance to echinocandins [58]	Fungal biocide resistance is not yet completely understood, being related to multiple defence mechanisms, including mutations, inducible efflux, exclusion or reduced access of antiseptic or disinfectant (chlorhexidine), enzymatic inactivation (formaldehyde) and phenotypic modulation (alcohol) [59,74] Virulence factors such as biofilm-forming capabilities and melanin further contribute to protection against biocides in fungi
	*Cryptococcus neoformans*	Cryptococcal meningitis, pulmonary cryptococcosis, cutaneous infections	Mutations in *ERG11*, overexpression of *ERG11* due to chromosome 1 duplication and upregulation of *AFR1* gene (encodes ABC transporter) confer resistance to azoles [75] Mutation in *ERG2* resulting in its inactivation, confers resistance to amphotericin b [76]	
	*Aspergillus niger*	Pulmonary aspergillosis, Aspergillus bronchitis, allergic bronchopulmonary aspergillosis (ABPA)	Azole resistance related to point mutations in *Cyp51A* gene, overexpression of *Cyp51A* gene and upregulation of efflux pumps [77]	
Viral	Human papillomavirus (HPV) (nonenveloped)	Cervical cancer	No treatment available	Nonenveloped viruses are more resistant to biocides, showing reduced susceptibility/resistance to lipophilic agents such as Qacs [13]
	Human immunodeficiency virus (HIV) (enveloped)	Acquired immunodeficiency syndrome (AIDS)	Drug resistance is caused by changes in the genetic structure of HIV that affect the ability of drugs (e.g., HAART) to block the replication of the virus [78]	Enveloped viruses are the least resistant to inactivation by biocides, where their lipid envelope is easily compromised by most disinfectants and antiseptics [13]
	Severe acute respiratory syndrome coronavirus 2 (SARS-CoV-2) (enveloped)	Respiratory illness	No treatment available	

## Data Availability

All data generated or analysed are openly available and included in this published article.
